# Focal ambient visual acuity stimulation improves vision in amblyopic children with stagnating BCVA—A retrospective study

**DOI:** 10.1111/aos.70137

**Published:** 2026-04-14

**Authors:** Kai Januschowski, Abdullah Salim, Anna Thonet, Spyridon Dimopoulos, Henrike Julich‐Haertel, Johann‐Peter Engels, Norbert Schulz, Stéphanie Langefeld, Rudolf Siegel, Susanne Hopf

**Affiliations:** ^1^ Mount St. Peter Eye Clinic Trier Germany; ^2^ University of Tuebingen Tuebingen Germany; ^3^ Medical Eye Research Xperts Institut (MerxI) Trier Germany; ^4^ Klaus Heimann Eye Research Institute (KHERI) Knappschaftsklinikum Saar GmbH, an der Klinik 10 Sulzbach Germany; ^5^ Ober Scharrer Group Eye Practice Gütersloh, Am Kolbeplatz Gütersloh Germany; ^6^ Eye Practice Rostock Rostock Germany; ^7^ Eye Practice Dr. Langefeld Trier Germany; ^8^ CISPA Helmholtz Center for Information Security Saarbrücken Germany; ^9^ University of Mainz Mainz Germany

**Keywords:** amblyopia treatment, Caterna vision training, digital amblyopia therapy, FAVAS, occlusion therapy, refractory amblyopia

## Abstract

**Purpose:**

The aim of this study was to investigate whether amblyopic children with stagnating best‐corrected visual acuity (BCVA) would benefit from an additional stimulation therapy—specifically focal ambient visual acuity stimulation (FAVAS) integrated into a computer game—compared to occlusion therapy alone.

**Methods:**

This retrospective, multicentre study analysed clinical records of 227 children with amblyopia showing stagnation of visual acuity despite at least 90 days of occlusion therapy. Analyses were performed on an eye‐based level. Eyes that had received FAVAS (Caterna Vision GmbH, Potsdam, Germany) alongside occlusion therapy were assigned to the FAVAS group, while eyes treated with occlusion therapy alone served as controls. Post hoc propensity score matching was applied. After matching, 105 patients (114 eyes; 57 eyes per group) remained for analysis. The primary endpoint was improvement in best corrected visual acuity (BCVA, logMAR); the secondary endpoint was change in stereo vision.

**Results:**

The FAVAS group showed a significant improvement in BCVA, with a mean gain of 0.15 logMAR (95% CI [0.11;0.18], *p* < 0.001), compared to a non‐significant improvement of 0.01 logMAR (95% CI [−0.02;0.04], *p* = 0.356) in the control group. No significant changes were observed in stereo vision in either group.

**Conclusion:**

The combination of occlusion therapy and FAVAS was superior to occlusion alone in amblyopic children with stagnating BCVA, yielding significant and clinically relevant improvements in visual acuity. These findings support the integration of digital stimulation therapy as an effective adjunct to conventional amblyopia treatment.

**Trial Registration:**

German Clinical Trials Register (DRKS): DRKS00030216.

## 1 INTRODUCTION

Amblyopia is a common disorder and a leading cause of visual impairment in children (Elflein et al., 2015). It arises from abnormal visual input during the critical period of visual development, leading to functional underdevelopment of the visual cortex. Anisometropia accounts for approx. 50% of cases, strabismus for 25%, while a combination of both is present in roughly one in six patients; other aetiologies are less frequent (Elflein, 2016).

Occlusion therapy with patching of the better eye after binocular refractive correction is the gold standard of care (BVA, 2010). First introduced by Sattler in the 1920s (Simonsz, 2009), this approach stimulates the amblyopic eye through forced use. Treatment outcomes depend on several factors, including age at initiation, severity of amblyopia, binocular function and fixation pattern, occlusion dose and compliance (Stewart et al., 2005). Among these, compliance is considered the most critical and modifiable factor.

Despite its efficacy, occlusion therapy has limitations. In many cases, especially in older children or those with a longer treatment history, visual improvement plateaus or regresses after initial gains (BVA, 2010). Furthermore, the psychosocial burden of wearing an eye patch can negatively affect adherence and emotional well‐being (Horwood et al., 2005). This underlines the need for additional therapeutic strategies when occlusion alone fails to deliver further improvement.

In recent years, binocular and stimulation‐based treatment approaches have gained increasing attention as alternatives or adjuncts to patching.

A recent randomised controlled trial has shown that supervised dichoptic video game‐based therapies can achieve visual acuity improvements comparable to occlusion therapy, while requiring substantially fewer treatment hours, particularly in older children (Kadhum et al., 2024). These findings are supported by a recent systematic review and meta‐analysis reporting no significant differences in visual acuity, stereoacuity, compliance or safety between dichoptic therapies and occlusion in paediatric amblyopia. However, most evidence derives from controlled, clinical‐based treatment settings (Halabi & Aldhahwani, 2025).

Many stimulation‐based approaches are not easily accessible and often exclude patching. To address these limitations, a home‐based, low‐barrier alternative that is combined with patching has been developed: Caterna Vision Training (Caterna Vision GmbH, Potsdam, Germany). This digital therapy uses focal ambient visual acuity stimulation (FAVAS) by integrating sinusoidally modulated circular grid patterns into the backgrounds of computer games. The method combines focused gaming with subtle ambient stimulation to engage the visual system, and it can be performed on a standard computer or tablet (≥ 10 inches) at home. Remote monitoring by ophthalmologists or orthoptists is supported.

The theoretical foundation of FAVAS is based on meridional amblyopia therapy principles. A randomised crossover study by Kämpf et al. demonstrated that moving grid patterns combined with occlusion significantly improved best corrected visual acuity (BCVA) in amblyopic children with astigmatism, particularly along the most ametropic meridian, while stationary grid patterns showed no significant effect. These findings suggest that dynamic visual stimulation may be critical for reactivating visual plasticity in meridional amblyopia (Kämpf et al., 2022). FAVAS may be effective in reactivating visual gains in children with stagnating visual development, yet broader real‐world data have been lacking. Notably, there is no clinical rationale to assume a different treatment response between unilateral and bilateral amblyopia for such stimulation‐based therapies. Therefore, it is hypothesised to benefit a broad range of amblyopic patients regardless of laterality.

The present study aims to evaluate the real‐world effectiveness of FAVAS in amblyopic children who showed stagnation of visual acuity after at least 90 days of occlusion therapy.

## 2 METHODS

### 2.1 Study design and setting

This two‐arm, retrospective, multicentre observational study aimed to evaluate the efficacy of focal ambient visual acuity stimulation (FAVAS) using the Caterna Vision Training system.

Patient data were collected between 01/01/2016 and 01/11/2023 across several participating centres: Eye Practice Rostock, Ophthalmology Group Practice Gütersloh, Mount St. Peter Eye Clinic, Eye Practice Dr. Langefeld and Colleagues in Trier, Eye Practice Dr. Kohler and Colleagues in Sulzbach and the University Eye Clinic of Tübingen. As a retrospective study, patients were assigned to groups retrospectively. To minimise bias from known confounding variables, propensity score matching was applied post hoc.

Propensity score matching is a well‐established method for adjusting confounding variables in the evaluation of healthcare data (Kuss et al., 2016) and is particularly appropriate for real‐world settings (IQWiG, 2020).

### 2.2 Ethical considerations

This study was approved by the Medical Ethical Committee of the State Chamber of Medicine of Rhineland Palatinate in Mainz, Germany (reference number 2022‐16377‐238678). The research adhered to the tenets of the Declaration of Helsinki.

### 2.3 Inclusion and exclusion criteria

Patients with the clinical diagnosis of amblyopia (ICD‐10 H53.0, amblyopia ex anopsia) were eligible for inclusion if they exhibited either a visual acuity difference between the eyes greater than 0.1 logMAR or a visual acuity of less than 0.1 logMAR in the worse eye (BVA, 2010). Eligible forms of amblyopia included refractive amblyopia, strabismic amblyopia or a mixed‐type of both forms. Refractive amblyopia is defined as decreased visual acuity caused by anisometropia or high bilateral refractive error in the absence of manifest strabismus. Strabismic amblyopia is defined as decreased visual acuity associated with manifest heterotropia. Both foveolar and eccentric fixation types were included, with a higher proportion of foveolar fixation expected due to the higher prevalence.

All children received full refractive correction prior to occlusion therapy. Occlusion therapy was initiated only after a stable refractive correction phase, defined as a stagnation of visual acuity improvement for a period of 3 to 6 months, in accordance with current clinical guidelines. Participants were required to be between 4 and 12 years of age and have completed at least 90 days of prescribed occlusion therapy, with stagnation of visual acuity improvement documented for a minimum of 1 month prior to initiating the 90‐day FAVAS or control treatment.

Exclusion criteria comprised patients with a diagnosis of learning disability, deprivation amblyopia (i.e. visual impairment due to organic causes) or other ophthalmological conditions such as anomalies of the cornea, lens, or retina. Patients with conditions affecting visual prognosis or a diagnosis of epilepsy (ICD‐10 G40) were also excluded. Individuals who had previously undergone or were currently undergoing concurrent therapies, such as stimulation therapy, including FAVAS, were not eligible.

If no suitable match was found for a patient during the propensity score matching, the patient was excluded from the final analysis.

### 2.4 Outcomes

The primary outcome was the change in best‐corrected visual acuity (BCVA, logMAR) from baseline (t0) to the end of FAVAS treatment (t1), assessed via subjective refraction with line optotypes. Data were originally recorded in decimal acuity (DIN/EN ISO) and converted using the formula: logMAR = −log10(decimal acuity).

The secondary outcome was the change in stereopsis, measured using either the Titmus or Lang test. The Lang test was used for younger or less cooperative children. The Titmus test was used in older children with sufficient cooperation.

### 2.5 Treatment protocols

All patients received routine clinical care, consisting of refractive correction before starting standard occlusion therapy. Patients who, after stagnation of BCVA under 90 days of occlusion,

- continued occlusion therapy for another 90 days were grouped into the control group.
- received 90 days of Caterna Vision Training in combination with occlusion were grouped into the FAVAS group.

The decision to initiate FAVAS therapy was made by treating physicians.

FAVAS therapy: Caterna Vision Training is a browser‐based therapy designed for children aged 4–12 years. Interactive games are overlaid with sinusoidally modulated circular grating in the background to stimulate the ambient field of vision while maintaining attention with a focal task. Sessions were conducted at home using a standard digital device. A minimum screen size of 10 inches, standard brightness settings and a viewing distance of approximately 60 cm were required; device calibration was not necessary. All sessions (frequency and duration) were automatically recorded by the software and reviewed remotely. Eye patching of the dominant eye was continued during the sessions.

### 2.6 Data collection and management

Data were extracted from patient records by trained personnel from the Mount St. Peter Eye Clinic and entered into a validated electronic case report form (eCRF, secuTrial® InteractiveSystems GmbH, Berlins). Five timepoints were defined:

- t‐2: Documentation of prior therapies (e.g. glasses, strabismus surgery).
- t‐1: Start of occlusion monotherapy.
- t0: Start of FAVAS period. FAVAS group began combination therapy (FAVAS plus occlusion), while the control group continued with occlusion alone. Baseline data collection.
- t1: End of therapy (90 days); primary and secondary outcomes assessed.
- t2: Follow‐up at 90 days after FAVAS to evaluate the persistence of treatment effects.

Time flexibility of ±14 days was accepted for clinical documentation. FAVAS group usage data (duration and frequency) were extracted from the Caterna software.

### 2.7 Propensity score matching and matching variables

Propensity score matching was used to match patients from the control and FAVAS groups based on covariates affecting visual acuity. Matching ensured that the groups were comparable, with the propensity score calculated using a generalised linear model and optimal pair matching. Various matching techniques were compared, and the final approach was chosen based on criteria such as positivity, overlap and balancedness (IQWiG, 2020).

The matching variables that were fully available included age, type of amblyopia and treatment duration (occlusion in hours per day), and were included in the propensity score matching. Fixation, refraction and squint angle were missing for many patients and thus not available as matching variables.

### 2.8 Statistical analysis

Primary analysis: Repeated measures ANOVA was used to compare BCVA changes between FAVAS and the control group. Interaction terms were included to evaluate treatment effects. Post hoc test using Welch *t*‐tests were applied for pairwise comparisons.

Secondary analysis: Changes in stereopsis were analysed descriptively. A significance threshold of *p* < 0.05 was used. All statistical analyses were performed using R version 4.2.1.

Effect modification: In order to further analyse the influence of age on the treatment effect, a hierarchical linear mixed‐effects model was conducted, including the three‐way interaction between age, group and time as fixed effects, along with the previously mentioned covariates. Patients were included as a random intercept.

### 2.9 Sample size

Based on prior studies (Kämpf et al., 2001, 2008, 2022; Kaupke, 2020), an expected difference in BCVA improvement of 0.17 (FAVAS) versus 0.07 (control) logMAR was assumed (SD = 0.17 for the difference between t0 and t1; expected effect size Cohen's *d* = 0.37). For a power of 80% and alpha = 0.05, 48 patients per group were required. To allow for a 20% dropout rate, 58 FAVAS participants were required. A control‐to‐intervention ratio of 3:1 (*n* = 174 controls) was chosen to optimise the propensity score matching (Jung et al., 2007).

## 3 RESULTS

### 3.1 Participants

A total of 291 participants were initially identified from clinical records in this study and documented in the secuTrial® software. However, 64 individuals (45 in the control group, 19 in the FAVAS group) were excluded due to failure to meet the inclusion criteria, resulting in a final sample size of 227 participants who were eligible for analysis. Of these, 171 participants formed the control group, while 56 participants were in the FAVAS group.

As 17 patients in the control group (9.7%) and 10 patients in the FAVAS group (19%) were bilaterally amblyopic, the second eye was equally included resulting in a sample of 188 eyes in the control group and 66 eyes in the FAVAS group. Further analysis and also the matching were carried out on the basis of the eyes and not the persons.

After propensity score matching, 55 individuals in the control group (57 eyes) and 50 individuals in the FAVAS group (57 eyes) remained. Thus, the final sample comprised 105 individuals (114 eyes).

Descriptive statistics and details of the propensity score matching procedure are provided in Table S1 and Figure S1.

### 3.2 Patient characteristics

Mean age in the sample of 105 participants was 6.8 years (*M* = 6.9, SD = 1.6 years in the FAVAS group and *M* = 6.7, SD = 1.7 years in the control group), with 51 (49%) being female. Duration of occlusion therapy was 4.8 months and 5.1 h per day (both as prescription). Statutory health insurance covered 83 children (89.2%). In the control group, 53 children wore glasses (two had missing data), and in the FAVAS group, 48 children wore glasses, but two did not. None of the children had a history of atropine treatment. Three children in the control group (6%) had undergone strabismus surgery.

Demographic characteristics of the FAVAS and control group are presented in Table 1.

**TABLE 1:** Demographic characteristics of the FAVAS and control group.

	Control group (*N* = 55 individuals)	FAVAS group (*N* = 50 individuals)	Total (*N* = 105 individuals)	*p*
Age (years)				0.574
Mean (SD)	6.7 (1.7)	6.9 (1.6)	6.9 (1.6)	
Range	4.0–10.8	4.3–10.4	4.0–10.8	
Diagnosis				0.972
Both forms of amblyopia	17 (31%)	15 (30%)	32 (31%)	
Refractive amblyopia	28 (51%)	25 (50%)	53 (51%)	
Strabismus amblyopia	10 (18%)	10 (20%)	20 (19%)	
Duration of prescription *T* = 0 (months)				0.151
Mean (SD)	3.2 (0.5)	6.5 (17.2)	4.8 (11.9)	
Range	3.0–5.0	3.0–90.0	3.0–90.0	
Sex				0.622
*N* missing	2	2	4	
Male	25 (46%)	25 (50%)	50 (48%)	
Female	28 (51%)	23 (46%)	51 (49%)	
Health insurance				0.057
*N* missing	10	2	12	
Statutory	43 (96%)	40 (83%)	83 (89%)	
Private	2 (4%)	8 (17%)	10 (11%)	

Data of descriptive statistics before and after propensity score matching are provided in Table S1 and Figure S1.

### 3.3 Main outcome

In the FAVAS group, logMAR BCVA improved from 0.28 (t0) to 0.14 (t1) to 0.11 (t2). In the control group, logMAR BCVA changed slightly from 0.27 (t0) to 0.26 (t1) to 0.28 (t2), as displayed in Table 2.

**TABLE 2:** Differences in logMAR BCVA between control and FAVAS group.

logMAR BCVA	Control group (*N* = 57 eyes)	FAVAS group (N = 57 eyes)	Difference in visual acuity between groups
t‐1			−0.02 (95% CI [−0.10,0.07]), *d* = −0.07
*N* missing	1	0	
Mean (SD)	0.26 (0.21)	0.27 (0.24)	
Range	0.00–1.10	0.00–1.00	
t0			−0.01 (95% CI [−0.10,0.07]), *d* = −0.06
Mean (SD)	0.27 (0.22)	0.28 (0.23)	
Range	0.00–1.00	0.10–1.22	
t1			0.12 (95% CI [0.04,0.20]), *d* = 0.58
Mean (SD)	0.26 (0.22)	0.14 (0.20)	
Range	0.00–1.00	0.00–1.10	
t2			0.18 (95% CI [0.09,0.26]), *d* = 0.87
*N* missing	9	8	
Mean (SD)	0.28 (0.24)	0.11 (0.19)	
Range	0.00–1.00	0.00–1.00	

LogMAR visual acuity improvement in the FAVAS group and changes in the control group are displayed in Figure 1.

**FIGURE 1:**
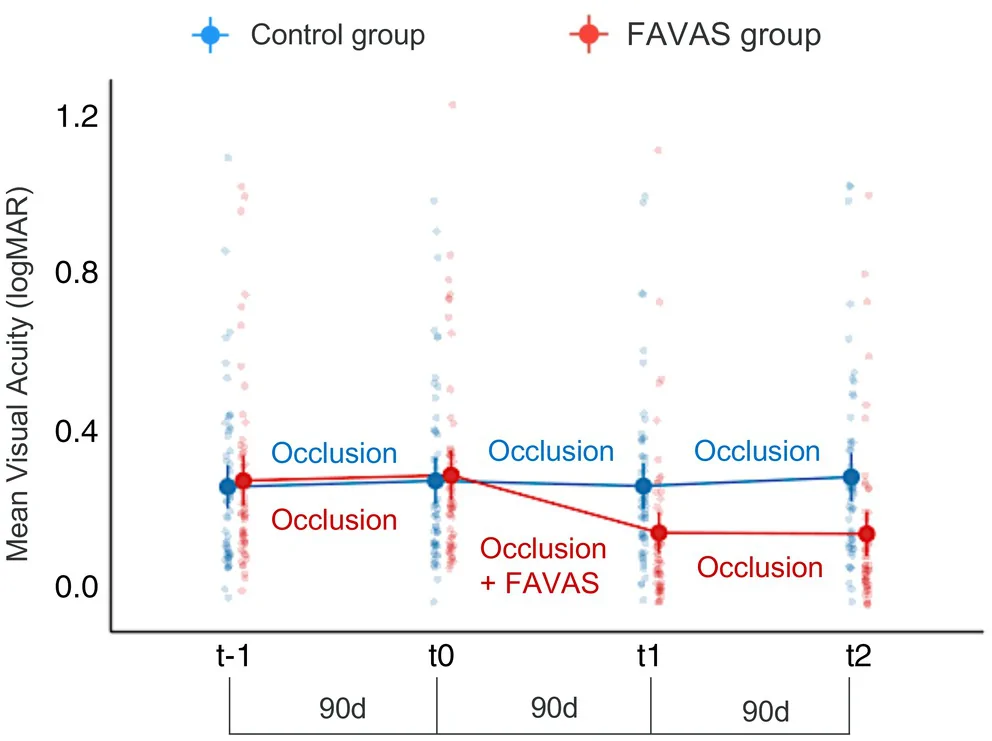
Chart of logMAR visual acuity in the FAVAS (red) and control (blue) group. The small dots in the background are the actual values of the individual persons. The thick dots and error bars correspond to the marginal mean values and their confidence interval.

We analysed visual acuity using an ANOVA with group (control vs. intervention) and time (t0 vs. t1) as within factors. The group was modelled as a within effect as the matching procedure resulted in pairs of participants. Based on the ANOVA, we calculated marginal means to conduct post hoc tests (*p*‐values corrected for multiple testing according to Holm). The post hoc tests revealed that the detected improvement from t0 to t1 in the FAVAS group was significant (*M* = 0.15 logMAR, 95% CI [0.11, 0.18], *p* < 0.001), whereas the change in the control group was not (*M* = 0.001 logMAR, 95% CI [−0.02, 0.04], *p* = 0.356).

### 3.4 Sensitivity analyses

Sensitivity analyses were conducted to assess the robustness of the primary findings. The main results remained consistent under various analytical approaches. Including age and refraction as covariates did not substantially alter the observed effects. In another analysis, baseline visual acuity exerted a moderate influence. Patients exhibiting poorer initial acuity demonstrated greater improvement in the FAVAS group; the group difference remained statistically significant. We repeated the main analysis but with the group as a between‐participant factor. This analysis yielded comparable results. Furthermore, the analysis of the entire sample, without matching, confirmed the robustness of the primary findings. A mixed model revealed minor age‐related differences, but the main findings were similar at t1. Analysis across all time points demonstrated sustained treatment effects at t2. In summary, the observed effect was consistently replicated across diverse modelling strategies, covariate adjustments and sample compositions.

### 3.5 Secondary outcome

At baseline (t0), 43% of children in the FAVAS group and 39% in the control group had stereo vision. At the follow‐up (t1), this increased to 49% and 42%, respectively. However, no significant difference was found between the groups over time. The findings are listed in Table 3.

**TABLE 3:** Distribution of stereo vision over time in both groups.

Stereo vision	Control group (*N* = 55 individuals)	FAVAS group (*N* = 50 individuals)
t‐1		
*N* missing	3	2
Yes	22 (42%)	19 (40%)
No	30 (58%)	29 (60%)
t0		
*N* missing	4	3
Yes	20 (39%)	20 (43%)
No	31 (61%)	27 (57%)
t1		
*N* missing	5	3
Yes	21 (42%)	23 (49%)
No	29 (58%)	24 (51%)
t2		
*N* missing	11	11
Yes	19 (43%)	18 (46%)
No	25 (57%)	21 (53%)

## 4 DISCUSSION

This retrospective study demonstrates that amblyopic children with stagnating visual acuity development can achieve a statistically significant and clinically relevant improvement in visual acuity through an additional 90‐day FAVAS therapy using Caterna, while stereovision is not improved. The structured data now available provide a scientifically robust basis for recommending the clinical use of FAVAS therapy for the first time in a large real‐life setting.

The study included patients from multiple ophthalmology centres, supporting the generalisability of findings to real‐world practice. The age of participating children was representative of children actively undergoing amblyopia treatment. The distribution of amblyopia types (refractive vs. strabismic) mirrors epidemiological findings; however, anisometropic and strabismic amblyopia appear to respond differently to amblyopia therapies, including digital approaches (Tang et al., 2025). Bilateral amblyopia was included and separately analysed. With a prevalence of 0.11% in the general population compared to 0.63% for unilateral cases (Xiao et al., 2022), the underrepresentation in our study is expected but does not undermine overall findings.

The study's therapy protocols were guideline‐based and standardised across participants, ensuring comparability between treatment arms (BVA, 2010). To further address potential confounding, propensity score matching was applied, a method widely accepted in health technology assessment (IQWiG, 2020).

Socioeconomic disparities, reflected for instance in insurance status, have known impacts on healthcare outcomes and access (Epping et al., 2021). To mitigate these effects, this study included a diverse patient population across multiple insurers and locations. Moreover, data collection over several years including during the COVID‐19 pandemic likely enhanced heterogeneity and reduced systematic bias. Therefore, the study setting allows for drawing conclusions.

The visual acuity gains observed are consistent with prior retrospective analyses (Kämpf et al., 2008) and prospective studies involving smaller cohorts, which reported BCVA improvement of 1.9 lines (SD 1.3) on the logMAR scale following short‐term FAVAS use (Kämpf et al., 2001, 2022).

Unlike binocular (dichoptic) systems that present different stimuli to each eye, FAVAS functions as a monocular training method: it separates foreground and background stimuli to the amblyopic eye while the better‐seeing eye is occluded. Unlike dichoptic therapies, it does not directly train binocular interaction, which likely explains the absence of a clinically relevant effect on stereopsis.

Despite this fundamental difference in treatment principle, comparisons with dichoptic digital therapies remain relevant, especially given recent meta‐analytic data indicating only slightly differing outcomes (0.16 logMAR improvement with dichoptic training versus 0.15 logMAR with monocular training) (Tang et al., 2025). Overall, our findings support the use of FAVAS as an effective adjunctive treatment to occlusion therapy, offering superior visual acuity outcomes compared to patching alone.

### 4.1 Limitations

Due to the retrospective study design, the results must be interpreted with caution. Potential sources of bias include the natural course of amblyopia, individual variations in therapy adherence and unrecorded external influences. Nevertheless, the course of amblyopia is well understood and generally not characterised by spontaneous improvement, particularly beyond the critical period (BVA, 2010; Kämpf, 2003; Kaupke, 2020). Daily adherence to occlusion therapy was not objectively tracked, introducing a limitation. Previous research indicates that parents tend to overreport adherence (Ihmig et al., 2022; Januschowski, Emmerich, et al., 2019; Januschowski, Rickmann, et al., 2019), similar to patterns observed in medication compliance studies.

Long‐term persistence of treatment effects beyond 90 days after FAVAS remains unclear.

## 5 CONCLUSION AND FUTURE PERSPECTIVE

The combination of FAVAS and occlusion therapy yielded significant and clinically relevant improvement in BCVA compared to occlusion alone. Our findings support the use of FAVAS as an adjunctive therapy in routine care settings with stagnant progress, while further RCTs are needed to confirm causality and long‐term benefits. Caterna Vision Training offers a low‐barrier, home‐based adjunctive therapy that may improve adherence and patient engagement. Future developments, such as the integration of streaming content, could further increase accessibility and motivation, especially in young patients.

## CONFLICT OF INTEREST STATEMENT

The authors declare no conflicts of interest.

## Supporting information


**Table S1.** Descriptive characteristics of the control and FAVAS group before and after matching.
**Figure S1.** Distribution of propensity scores in the control and FAVAS group before and after matching. The points represent the empirically available propensity scores.
