# Supplementary material for: Focal ambient visual acuity stimulation improves vision in amblyopic children with stagnating BCVA—A retrospective study
Source: Acta Ophthalmol. 2026 Apr 14;104(7):811–7. doi: 10.1111/aos.70137 (PMC13651917; doi:10.1111/aos.70137)
Supplement: Supplementary file 1 [file AOS-104-811-s001.pdf]

Supplementary Material

Table 1: Descriptive characteristics of the control and FAVAS group before and after matching.

	Before Matching		After matching		
	**CONTROLS (n=171, eyes=188)**	**FAVAS (n=56, eyes=66)**	**CONTROLS (n=55, eyes=57)**	**FAVAS (n=50, eyes=57)**	**FAVAS excluded (n=6, eyes=9)**
**AGE (months)**					
Mean (SD)	78.0 (20.5)	86.3 (20.4)	80.8 (20.7)	83.0 (18.7)	113.8 (10.8)
Range	48.0 - 135.0	51.0 - 132.0	48.0 - 129.0	51.0 - 125.0	99.0 - 132.0
**EYE SIDE**					
OS	103 (60.2%)	31 (55.4%)	32 (58.2%)	26 (52.0%)	5 (83.3%)
OD	68 (39.8%)	25 (44.6%)	23 (41.8%)	24 (48.0%)	1 (16.7%)
**DURATION OF PRESCRIPTION (months)**					
Mean (SD)	3.1 (0.4)	6.2 (16.3)	3.2 (0.5)	6.6 (17.2)	3.0 (0.0)
Range	3.0 - 5.0	3.0 - 90.0	3.0 - 5.0	3.0 - 90.0	3.0 - 3.0
**HOURS OF OCCLUSION PER DAY**					
Mean (SD)	4.9 (1.5)	5.4 (2.3)	5.2 (1.7)	5.1 (2.2)	8.0 (0.0)
Range	1.0 - 8.0	1.0 - 8.0	2.0 - 8.0	1.0 - 8.0	8.0 - 8.0
**PERIOD OF USE (MINUTES)**					
N missing	171	0	55	0	0
Mean (SD)	NA	3285 (1319)	NA	3184 (1299)	4127 (1281)
Range	NA	468 - 6952	NA	468 - 6952	2495 - 5793
**DIAGNOSIS**					
Both forms of amblyopia	47 (27.5%)	21 (37.5%)	17 (30.9%)	15 (30.0%)	6 (100.0%)
Refraktive amblyopia	79 (46.2%)	25 (44.6%)	28 (50.9%)	25 (50.0%)	0 (0%)
Strabismus amblyopia	45 (26.3%)	10 (17.9%)	10 (18.2%)	10 (20.0%)	0 (00%)
**STEREO VISION (t0)**					
N missing	10	4	4	3	1
Ja	60 (37.3%)	22 (42.3%)	20 (39.2%)	20 (42.6%)	2 (40.0%)
Nein	101 (62.7%)	30 (57.7%)	31 (60.8%)	27 (57.4%)	3 (60.0%)
**BCVA amblyopic eye t=0**					

	Before Matching		After matching		
	**CONTROLS (n=171, eyes=188)**	**FAVAS (n=56, eyes=66)**	**CONTROLS (n=55, eyes=57)**	**FAVAS (n=50, eyes=57)**	**FAVAS excluded (n=6, eyes=9)**
Mean (SD)	0.28 (0.22)	0.28 (0.23)	0.27 (0.23)	0.29 (0.24)	0.22 (0.10)
Range	-0.00 - 1.00	0.10 - 1.22	-0.00 - 1.00	0.10 - 1.22	0.10 - 0.40
Range	-0.000 - 1.000	0.046 - 1.222	-0.000 - 1.000	0.046 - 1.222	0.097 - 0.222
**GRADE OF FIXATION t=0**					
N missing	74	44	27	39	5
Mean (SD)	2.12 (5.04)	1.42 (3.37)	1.39 (3.70)	1.55 (3.50)	0.00 (NA)
Range	0.00 - 20.00	0.00 - 10.00	0.00 - 15.00	0.00 - 10.00	0.00 - 0.00
**GRADE OF STRABISMUS OS t=0**					
N missing	127	52	35	46	6
Mean (SD)	6.32 (11.46)	6.50 (9.04)	4.15 (9.98)	6.50 (9.04)	NA
Range	-25.00 - 30.00	1.00 - 20.00	-17.00 - 20.00	1.00 - 20.00	NA
**GRADE OF STRABISMUS OD t0**					
N missing	131	46	44	41	5
Mean (SD)	7.83 (7.46)	4.60 (4.48)	9.27 (6.12)	5.00 (4.56)	1.00 (NA)
Range	-7.00 - 25.00	1.00 - 15.00	2.00 - 20.00	1.00 - 15.00	1.00 - 1.00

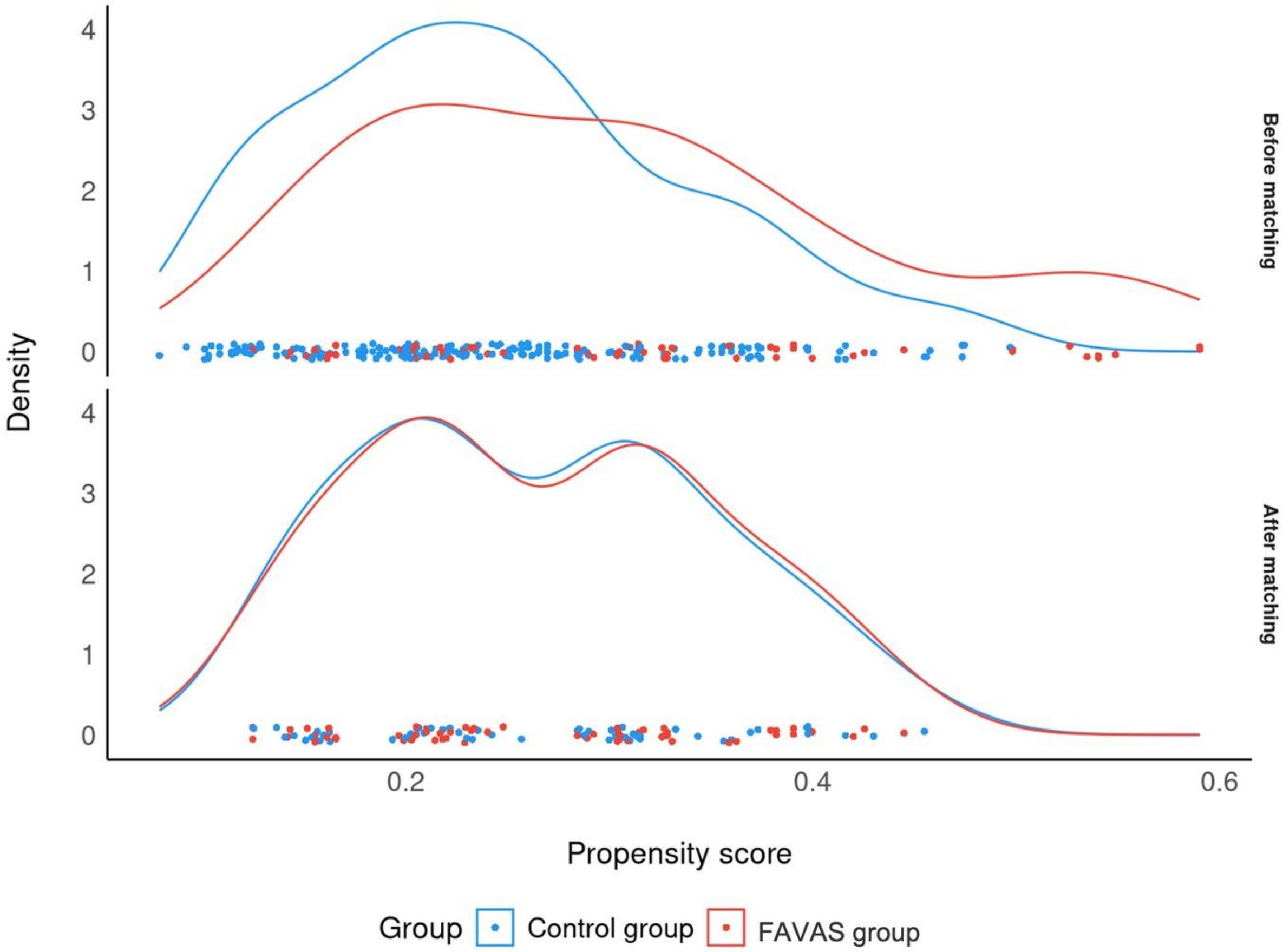

Figure 1: Distribution of propensity scores in the control and FAVAS group before and after matching. The points represent the empirically available propensity scores.
